# Curcumin alleviates orofacial allodynia and improves cognitive impairment via regulating hippocampal synaptic plasticity in a mouse model of trigeminal neuralgia

**DOI:** 10.18632/aging.204984

**Published:** 2023-08-25

**Authors:** Hong-Wei Zhi, Yu-Zhi Jia, Huai-Qian Bo, Hai-Tao Li, Si-Shuo Zhang, Ya-Han Wang, Jie Yang, Ming-Zhe Hu, Hong-Yun Wu, Wen-Qiang Cui, Xiang-Dong Xu

**Affiliations:** 1 Department of Neurology, Affiliated Hospital of Shandong University of Traditional Chinese Medicine, Jinan, Shandong, PR China; 2 First College of Clinical Medicine, Shandong University of Traditional Chinese Medicine, Jinan, Shandong, PR China; 3 Xiyuan Hospital, China Academy of Chinese Medical Sciences, Beijing, PR China; 4 Experimental Center, Affiliated Hospital of Shandong University of Traditional Chinese Medicine, Jinan, Shandong, PR China

**Keywords:** curcumin, chronic pain, cognitive impairment, synaptic plasticity, hippocampal CA1 region

## Abstract

Objective: Cognitive impairment, one of the most prevalent complications of trigeminal neuralgia, is troubling for patients and clinicians due to limited therapeutic options. Curcumin shows antinociception and neuroprotection pharmacologically, suggesting that it may have therapeutic effect on this complication. This study aimed to investigate whether curcumin alleviates orofacial allodynia and improves cognitive impairment by regulating hippocampal CA1 region synaptic plasticity in trigeminal neuralgia.

Methods: A mouse model of trigeminal neuralgia was established by partially transecting the infraorbital nerve (pT-ION). Curcumin was administered by gavage twice daily for 14 days. Nociceptive thresholds were measured using the von Frey and acetone test, and the cognitive functions were evaluated using the Morris water maze test. Dendritic spines and synaptic ultrastructures in the hippocampal CA1 area were observed by Golgi staining and transmission electron microscopy.

Results: Curcumin intervention increased the mechanical and cold pain thresholds of models. It decreased the escape latency and distance to the platform and increased the number of platform crossings and dwell time in the target quadrant of models, and improved spatial learning and memory deficits. Furthermore, it partially restored the disorder of the density and proportion of dendritic spines and the abnormal density and structure of synapses in the hippocampal CA1 region of models.

Conclusion: Curcumin alleviates abnormal orofacial pain and cognitive impairment in pT-ION mice by a mechanism that may be related to the synaptic plasticity of hippocampal CA1, suggesting that curcumin is a potential strategy for repairing cognitive dysfunction under long-term neuropathic pain conditions.

## INTRODUCTION

Characterized by recrudescent electric shock-like orofacial pain [1], trigeminal neuralgia (TN) is regarded as one of the most painful conditions known to humankind. Severe pain affects quality of life, both physically and mentally [2]. Moreover, one of the most common complications of TN is cognitive impairment [3, 4], which seriously affects patients’ daily functions and activities.

The first-line therapy for TN is the administration of carbamazepine or oxcarbazepine [5]. However, this treatment has many side effects and a high probability of recurrence [6], which warrants the search for better candidate treatment drugs. Curcumin is a polyphenol extracted from the root of curcuma longa, and its medicinal use has aroused widespread interest. Cumulative studies have demonstrated the therapeutic effects of curcumin in multiple diseases of the nervous system such as depression [7], Alzheimer’s disease [8], epilepsy [9], and, above all, chronic pain [10]. Notably, a high dose of curcumin is still safe for humans [11]. Studies have demonstrated that curcumin can alleviate chronic pain and improve cognitive impairment by providing hippocampal neuroprotection in rat models with sciatic nerve constriction and cobra venom-induced trigeminal neuralgia [10, 12]. However, it is not clear whether curcumin can attenuate trigeminal neuralgia and related cognitive impairment in mice, and whether this occurs through a hippocampal neuroprotective mechanism.

The hippocampus, consisting of the dentate gyrus (DG) and cornu ammonis (CA), is integral to the so-called “cognitive map”, which enables organisms to explore and adapt to the environment [13]. In addition, the hippocampus plays an important role in regulating allodynia and its accompanying cognitive impairment [14, 15]. It has been reported that CA1 and DG of the hippocampus are involved in the pain response by mediating the activation of 5-hydroxytryptamine receptors [16]. The impairment of spatial learning and memory function in chronic neuropathic pain model rats may occur by increasing GABA concentration and reducing glutamate and BDNF levels in the hippocampal CA1 region [17]. Synaptic plasticity is the cellular basis for learning and memory. It affects the formation of synaptic connections and neural circuits, resulting in gain or loss of action and function [18]. In painful conditions, synaptic plasticity can adaptively change to reduce pain by reversing long-term potentiation and structural changes in CA1 [19–21]. The disruption of hippocampal NMDRs can disturb long-term potentiation (LTP), and result in memory deficits [22]. Thus, regulating hippocampal synaptic plasticity may be key to treating chronic pain and related cognitive impairments.

Therefore, we conducted the present study to investigate whether curcumin could alleviate orofacial allodynia and improve cognitive impairment by regulating hippocampal synaptic plasticity in a mouse model of trigeminal neuralgia.

## MATERIALS AND METHODS

### Animals

Healthy male C57BL/6 mice (7–9 weeks old, 20–25 g) were purchased from the Beijing Vital River Laboratory Animal Technology Co., Ltd., Beijing, China. The sample size of the animals was determined based on previous studies [23]. Mice were placed in separate cages (22 ± 1°C, 60% humidity, under a 12-hour light/dark cycle) and allowed to eat and drink freely. All animal experiments in this study were approved by the Ethics Committee of the Affiliated Hospital of Shandong University of Traditional Chinese Medicine (Jinan, China) and conducted according to the guidelines of the International Association for the Study of Pain. Every effort was made to minimize the number of animals used and their pain.

### pT-ION model

**Figure 1:**
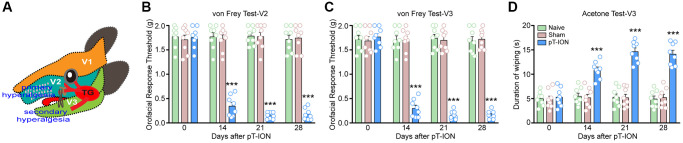
**The time course of pT-ION induced orofacial allodynia.** (**A**) The schematic diagram of orofacial branch of the trigeminal nerve and pT-ION surgery. (**B**) and (**C**) showed the primary and secondary mechanical hyperalgesia induced by pT-ION in the ipsilateral facial V2 and V3 area, respectively. (**D**) showed the secondary cold allodynia in the ipsilateral facial V3 area. *N* = 8 mice/group. ^***^P < 0.001 vs. naive group, two-way ANOVA and Tukey’s test.

The mouse model of trigeminal neuralgia was established by partially transecting the infraorbital nerve (pT-ION) as in our previous experiments [24]. Briefly, the pT-ION or sham surgery group was anesthetized with sodium pentobarbital (50 mg/kg, intraperitoneal injection), the mice were then placed on a surgical pad and fixed in a head-down supine position. The mouth of the mouse was opened and a 5-mm long incision was made on the palatal-buccal mucosa to the left of the first molar. The nerves were gently separated using a pair of blunt glass rods. The deep branch of the nerve was ligated with sterile lamb’s intestinal thread (4.0, BD171001, Boda Co., Shandong, China), its distal end was cut with surgical scissors, and approximately 1 mm of the nerve fibers were excised to prevent regeneration (Figure 1A). The wound was sealed with tissue glue and the animal was allowed to recover from anesthesia on a warm blanket after the surgery. The surgery for the sham group exposed only the left infraorbital nerve but did not ligate and cut the nerve. All procedures were performed under aseptic conditions and no serious infections or postoperative complications occurred. The naive group was not administered any treatment. Three mice were excluded from the follow-up experiment because of wasting, abnormal movement, and poor mental status.

### Assessment of allodynia behavior

The von Frey test was used to assess the neurological mechanical abnormalities of pain, as described in a previous study [24]. Oral mechanical stimulation threshold of mice was measured before surgery and on post-surgical days 14, 21, and 28. In order to reduce the impact of restraint of mice during testing, mice were allowed to acclimate to the test environment (a box (7 × 7 × 10 cm^3^) made of black wire mesh) for at least 10 min, a range of von Frey filament (Stoelting, Niagara Falls, NY, USA) bending forces (0.07–2.0 g) was applied to the skin of the V2 and V3 regions (pT-ION ipsilateral, Figure 1A), and behavioral responses were observed. Each von Frey applied filaments five times at intervals of several seconds. A positive response was defined as a quick withdrawal of the head after the von Frey filament. The bending force (g) of the von Frey filament at which three positive responses were observed out of five stimulations was defined as the threshold.

The acetone test was used to assess neurogenic cold pain hypersensitivity response [24]. Briefly, mice were allowed to acclimate to the test environment for at least 10 min, and a diluted 50 μL solution of acetone (90% concentration) was dropped onto the skin of the V3 region of the mouse (ipsilateral to the pT-ION) using a syringe (Hamilton, Reno, NV, USA) attached to a specific 25-gauge needle. The positive response (rubbing or scratching the skin of the V3 region) was monitored, and the cut-off time was set to 2 min. The baseline acetone values were measured under the same experimental settings prior to the experiment. To avoid injury from acetone entering the eyes of mice, we only tested the secondary cold nociception in the V3 region.

### Morris water maze test

The Morris water maze test (MWMT) was administered to assess the learning and spatial memory abilities of mice after neuropathic pain. After the 24th postoperative day, a 5-day MWMT was started, which consisted of orientation cruising and spatial exploration training. We prepared a circular pool (120 cm in diameter and 40 cm in height) divided into four quadrants using MWMT, and the escape platform (8 cm in diameter) was placed in the second quadrant with the center of the platform (22.5 cm) from the pool wall. Water (depth 30 cm, temperature 22 ± 1°C) was placed at the beginning of the experiment. The escape platform was submerged underwater for 1.5 cm, and edible white stain was added to the water to facilitate the video recording apparatus to produce contrasting colors and record the trajectory of the mice’s actions. Days 1–4 were for orientation cruising training, and the mice were subjected to acquisition tests at the same time each day, with each mouse gently placed into the pool facing the wall. If the mice could not find the platform within 60 s, they were placed in the platform position for 10 s and were allowed to perform spatial learning and memory. On day 5, the escape platform was removed from the pool and the mice were placed in the quadrant opposite to the platform position and allowed to swim freely for 60 s. The mice were monitored and recorded on video using a camera placed above the pool and analyzed using the ZS-001 video analysis system (Beijing Zhongshidichuang Science and Technology Development Co., LTD, Beijing, China). The escape latency, distance from the platform, number of times the mice crossed the platform, and time spent in the target platform quadrant were recorded. After all the tests, the mice were thoroughly dried with a hair dryer and returned to their cages. The MWMT was performed by two researchers who were blinded to the group assignments.

### Curcumin solution and dosage

Curcumin (C7727, total purity >94.0%) was purchased from Sigma-Aldrich Trading Co (Shanghai, China). Sodium carboxymethyl cellulose (CMC-Na) (C8621, viscosity 800–1200) was purchased from Beijing Solarbio Science and Technology Co. (Beijing, China). As curcumin is insoluble in water, we chose CMC-Na (a food additive usually used as a viscosity modifier) to improve its solubility and increase its bioavailability. Curcumin powder was poured into 0.5% CMC-Na solution and dissolved under magnetic stirring for 10 min. Curcumin (100 mg/kg/day) was administered to the mice intragastrically twice daily (once in the morning and the evening) for 14 days after the 14th postoperative day.

### The Golgi staining

The Golgi staining kit (FD Neuro Technologies, Inc., Columbia, MD, USA) and A solution : B solution (1:1) was mixed well for one day in advance, stored at room temperature, and protected from light. After the mice were anesthetized and executed, intact brain tissues were taken and put into an AB mixture (tissue: fixative = 1:5), and the solution was changed once at 24 h. The brain tissues were fixed for 14 days at 4°C and protected from light. After 14 days, the brain tissues were transferred to solution C and stored at 4°C for seven days. Brain hippocampal tissues were removed for sectioning (100 μm) and then placed in solutions D and E for 10 min for restaining (solution D, E: distilled water = 1:1:2). Tissue sections were rinsed twice in distilled water and then dehydrated with different gradients of ethanol (50%, 75%, and 95%) for 5 min each, rinsed with xylene, sealed with resin glue, and stored in the dark. The sections were observed under a microscope (Leica DM6000B, Germany). Images were reviewed, and dendritic spine density was analyzed using ImageJ software.

### Transmission electron microscopy (TEM)

Transmission electron microscopy (TEM) was applied to observe the morphological features of synapses, including synaptic density, thickness of the postsynaptic dense zone (PSD), length of the active zone, width of the synaptic cleft, and curvature of the synaptic interface. The mice were perfused with 0.9% saline, and fresh brain tissue was fixed in a 2% paraformaldehyde-glutaraldehyde solution. The hippocampal region of the brain tissue was quickly excised and placed in a new fixative solution overnight, washed three times with 0.1 mol/LPBS, and then fixed in 1% osmium tetroxide, and washed three more times. Subsequently, the samples were dehydrated using graded ethanol and acetone solutions. Hippocampal tissues were serially sectioned using an ultrathin sectioning machine at a thickness of 100 nm per slice, embedded in Eponate 812 medium (90529-77-4, Structure Probe, Inc., West Chester, PA, USA), and stored overnight. The sections were stained with 2% uranyl acetate for 30 min and 6% lead citrate for 5–10 min. The sections were observed using TEM (Hitachi Limited, Japan), and the images were analyzed using ImageJ software.

### Statistical analysis

Data are presented as the mean ± SEM. GraphPad Prism 6 (GraphPad Software Inc., San Diego, CA, USA) was administered for the statistical analysis of data. Two-way repeated-measures analysis of variance (ANOVA) followed by Tukey’s multiple comparison test was used to analyze the time course of mechanical and cold allodynia. One-way ANOVA followed by Dunnett’s test or Tukey’s test was applied to analyze the statistical significance of differences between groups. *P* < 0.05 was considered statistically significant.

### Data sharing statement

The data used to support the findings of this study are available from the corresponding author upon request.

## RESULTS

### pT-ION induces unilateral orofacial nociceptive hypersensitivity and cognitive impairment in mice

We assessed the abnormal nociceptive behavior of the mice using the von Frey and acetone test. Results showed no significant difference in the basal mechanical and cold pain thresholds of the groups (*P* > 0.05, Figure 1B–1D). There were no significant differences in mechanical and cold pain thresholds at each time point between the naive and sham groups (*P* > 0.05, Figure 1B–1D). However, mice in the pT-ION group showed reduced mechanical pain thresholds in the skin of V2 (two-way ANOVA and Tukey’s test, F_2,21_ = 110.00, *P* < 0.0001) and V3 (two-way ANOVA and Tukey’s test, F_2,21_ = 107.60, *P* < 0.0001) regions on days 14–28 after modeling (Figure 1B, 1C). The results of the acetone test showed that the duration of orofacial wiping in the V3 region was increased in the pT-ION group on day 14 after modeling compared to the naive group and continued until day 28 (two-way ANOVA and Tukey’s test, F_2,21_ = 61.47, *P* < 0.0001, Figure 1D). These findings suggest that pT-ION can cause mechanical nociceptive hypersensitivity as well as cold pain hypersensitivity in mice and that we successfully established a peripheral neuropathic pain model.

**Figure 2:**
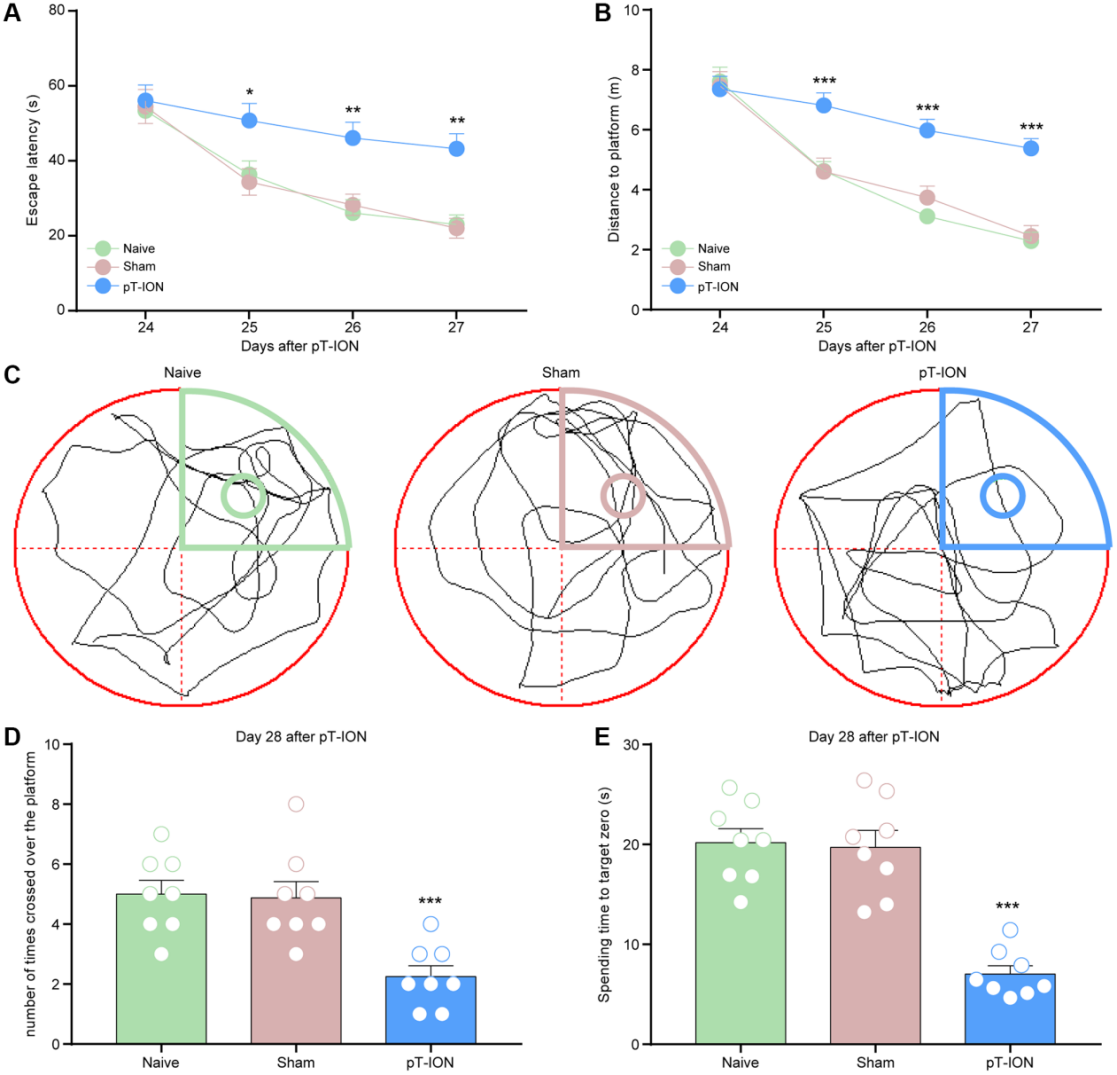
**The learning and memory impairment induced pT-ION mice.** The training trials were performed for 4 consecutive days (days 24, 25, 26, and 27 after pT-ION). The probe test was performed after the training (day 28 after pT-ION). (**A**) Time to reach the hidden platform. (**B**) Total swimming distance during the trial. (**C**) Tracks of the mice in the probe test on the fifth day. (**D**) Number of times the mice crossed over the platform. (**E**) Spending time to target zone area. *N* = 8 mice/group. ^*^P < 0.05, ^**^P < 0.01 and ^***^P < 0.001 vs. naive group, (**A**, **B**): two-way ANOVA and Tukey’s test, (**C**–**E**): one-way ANOVA and Dunnett test.

To investigate whether pT-ION mice showed spatial learning and memory dysfunction, a Morris water maze experiment was performed on postoperative days 24–28, and we found no statistical difference in escape latency and distance to platform between groups on day 24 (*P* > 0.05, Figure 2A, 2B). Compared with the naive group, the pT-ION group showed increased escape latency (two-way ANOVA and Tukey’s test, F_2,21_ = 17.69, *P* < 0.0001, Figure 2A) and distance to the platform (two-way ANOVA and Tukey’s test, F_2,21_ = 9.31, *P* = 0.0013, Figure 2B) on day 25–27, and decreased number of platform crossings (one-way ANOVA and Dunnett test, F = 11.14, *P* = 0.0005) and target platform quadrant dwell time (one-way ANOVA and Dunnett test, F = 30.31, *P* < 0.0001) on day 28 (Figure 2C–2E). These results indicate that cognitive impairment is caused by peripheral nerve injuries.

### Curcumin alleviates pT-ION-induced unilateral orofacial pain and cognitive impairment in mice

**Figure 3:**
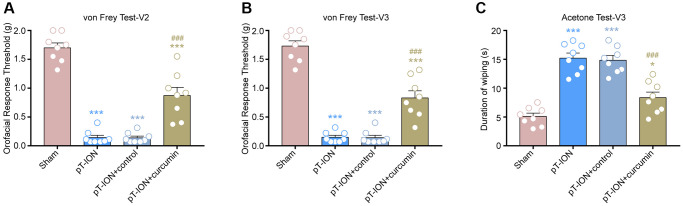
**The effect of curcumin on orofacial allodynia induced by pT-ION.** (**A**) The primary mechanical hyperalgesia in area V2. (**B**) The secondary mechanical hyperalgesia in area V3. (**C**) The secondary cold allodynia in area V3. *N* = 8 mice/group. ^*^P < 0.05 and ^***^P < 0.001 vs. sham group; ^###^*P* < 0.001 vs. pT-ION group, one-way ANOVA and Tukey’s test.

**Figure 4:**
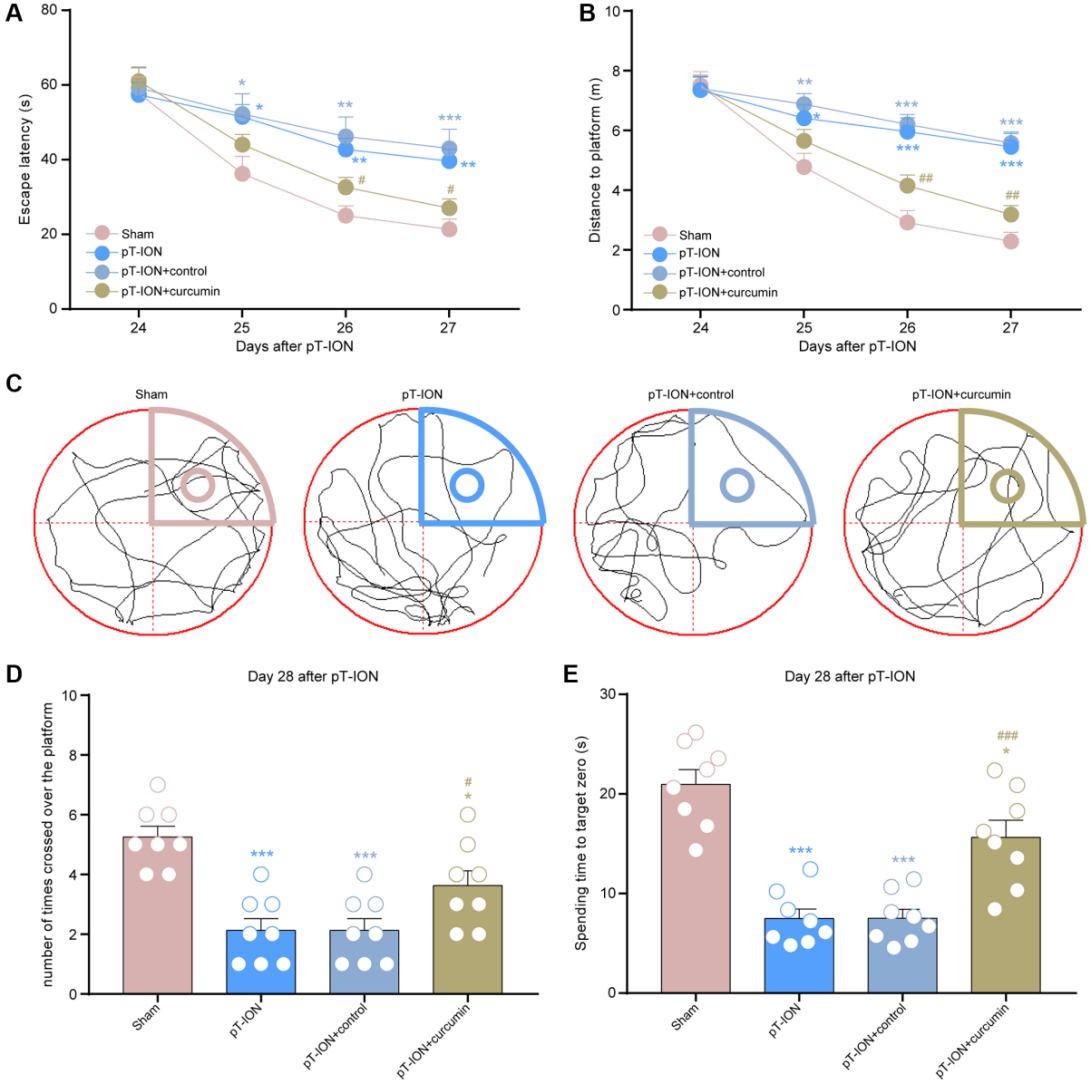
**The effect of curcumin on learning and memory impairment in pT-ION mice.** (**A**) Time to reach the hidden platform. (**B**) Total swimming distance during the trial. (**C**) Tracks of the mice in the probe test. (**D**) Number of times the mice crossed over the platform. (**E**) Spending time to target zone area. *N* = 8 mice/group. ^*^P < 0.05, ^**^P < 0.01 and ^***^P < 0.001 vs. sham group; ^#^*P* < 0.05, ^##^*P* < 0.01 and ^###^*P* < 0.001 vs. pT-ION group, (**A**, **B**): two-way ANOVA and Tukey’s test, (**C**–**E**): one-way ANOVA and Tukey’s test.

From days 14–28, mice were administered curcumin by gavage for 14 consecutive days. The von Frey and acetone tests were performed on the 28th day after modeling to observe abnormal orofacial nociception in mice. The results showed that the mechanical (V2: one-way ANOVA and Tukey’s test, F = 73.67, *P* < 0.0001; V3: one-way ANOVA and Tukey’s test, F = 87.44, *P* < 0.0001) and cold pain (one-way ANOVA and Tukey’s test, F = 36.41, *P* < 0.0001) thresholds of the pT-ION + curcumin group increased, while those of the pT-ION + control group were not significantly different from those of the pT-ION group (Figure 3A–3C). The results of the MWMT showed that the pT-ION+ curcumin group had improved pT-ION-induced cognitive impairment compared with the pT-ION and pT-ION + control groups. The escape latency (two-way ANOVA and Tukey’s test, F_3,28_ = 3.365, *P* = 0.0325, Figure 4A) and distance to the target platform (two-way ANOVA and Tukey’s test, F_3,28_ = 6.914, *P* = 0.0013, Figure 4B) of the pT-ION + curcumin group significantly decreased on days 26 and 27, while both the number of times crossing the platform (one-way ANOVA and Tukey’s test, F = 12.73, *P* < 0.0001) and spending time to the target zone (one-way ANOVA and Tukey’s test, F = 25.61, *P* < 0.0001) significantly increased on day 28 (Figure 4C–4E).

### Curcumin increased the density of dendritic spine and mushroom-type dendritic spine in the hippocampus CA1 of pT-ION mice

**Figure 5:**
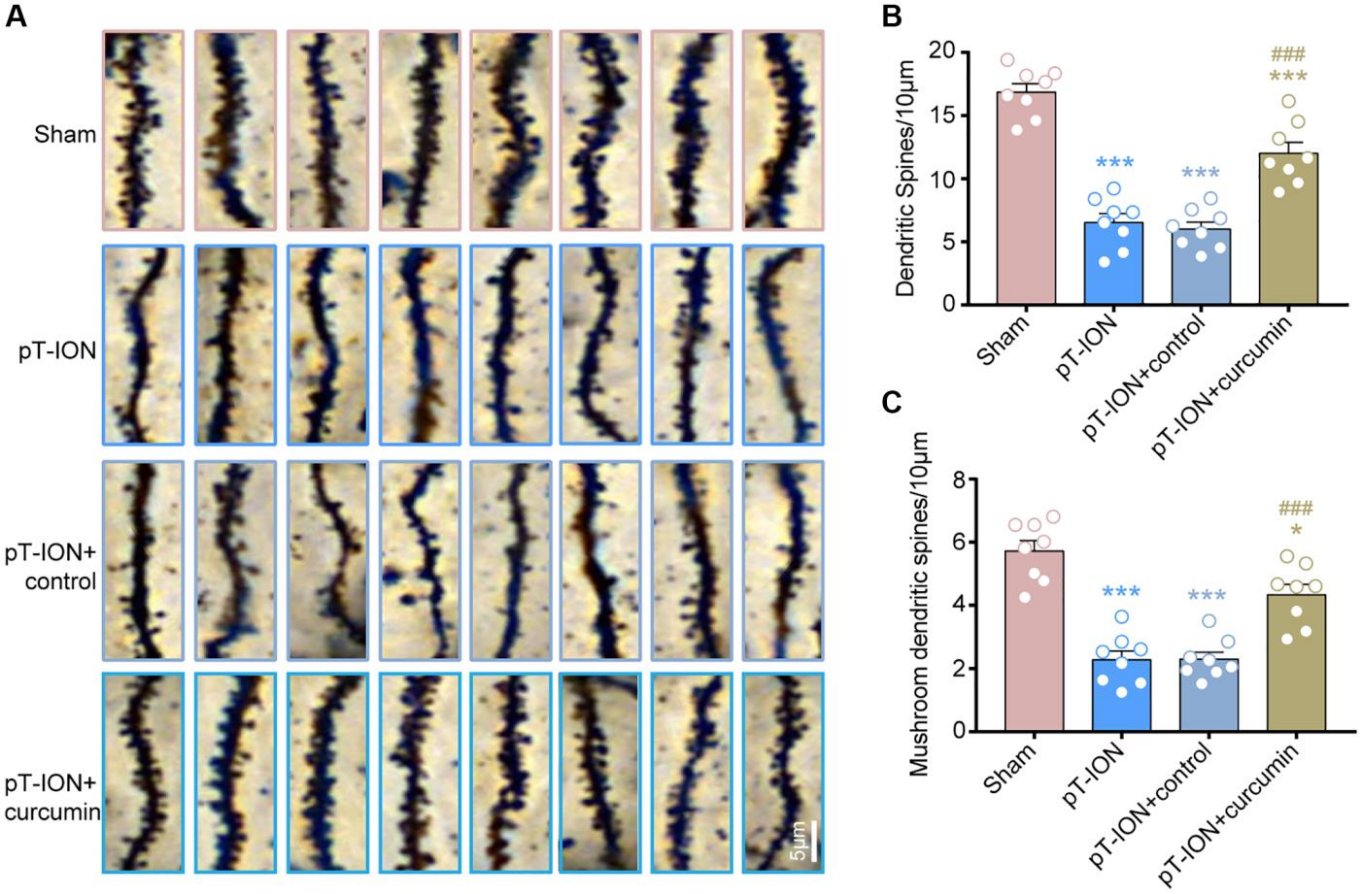
**The role of curcumin on the decrease of dendritic spine density induced by pT-ION in the mice hippocampal CA1 region.** (**A**) The representative Golgi-Cox-staining images showing the density and morphology of dendritic spines in the cone cell layer of hippocampal CA1 region in each group (scale bar: 5 μm). (**B**) The analysis of total dendritic spine density in each group. (**C**) The analysis of mushroom-type dendritic spine density in each group. *N* = 8 mice/group. ^*^P < 0.05 and ^***^P < 0.001 vs. sham group; ^###^*P* < 0.001 vs. pT-ION group, one-way ANOVA and Tukey’s test.

We analyzed dendritic spine density and the percentage of each type of dendritic spine in the hippocampal CA1 using Golgi staining. The results showed that the dendritic spine density was significantly reduced in the pT-ION (one-way ANOVA and Tukey’s test, *P* < 0.0001) and pT-ION + control (one-way ANOVA and Tukey’s test, *P* < 0.0001) groups compared with the sham group, and the density of mushroom-type dendritic spines in CA1 neurons was significantly reduced in the pT-ION (one-way ANOVA and Tukey’s test, *P* < 0.0001) and pT-ION + control (one-way ANOVA and Tukey’s test, *P* < 0.0001) groups compared with the sham group (Figure 5A–5C). These results indicate that pT-ION induced a significant loss of dendritic spines and reduced synaptic plasticity. However, the density of dendritic spines and mushroom-type dendritic spines in the pT-ION + curcumin group significantly increased compared with the pT-ION group (one-way ANOVA and Tukey’s test, *P* < 0.0001, Figure 5A–5C), indicating that curcumin treatment could increase the density of dendritic spines, regulate the proportion of dendritic spines, and alleviate the synaptic damage of hippocampal CA1 neurons.

### Curcumin promotes normalization of synapse number and structure in pT-ION mice

**Figure 6:**
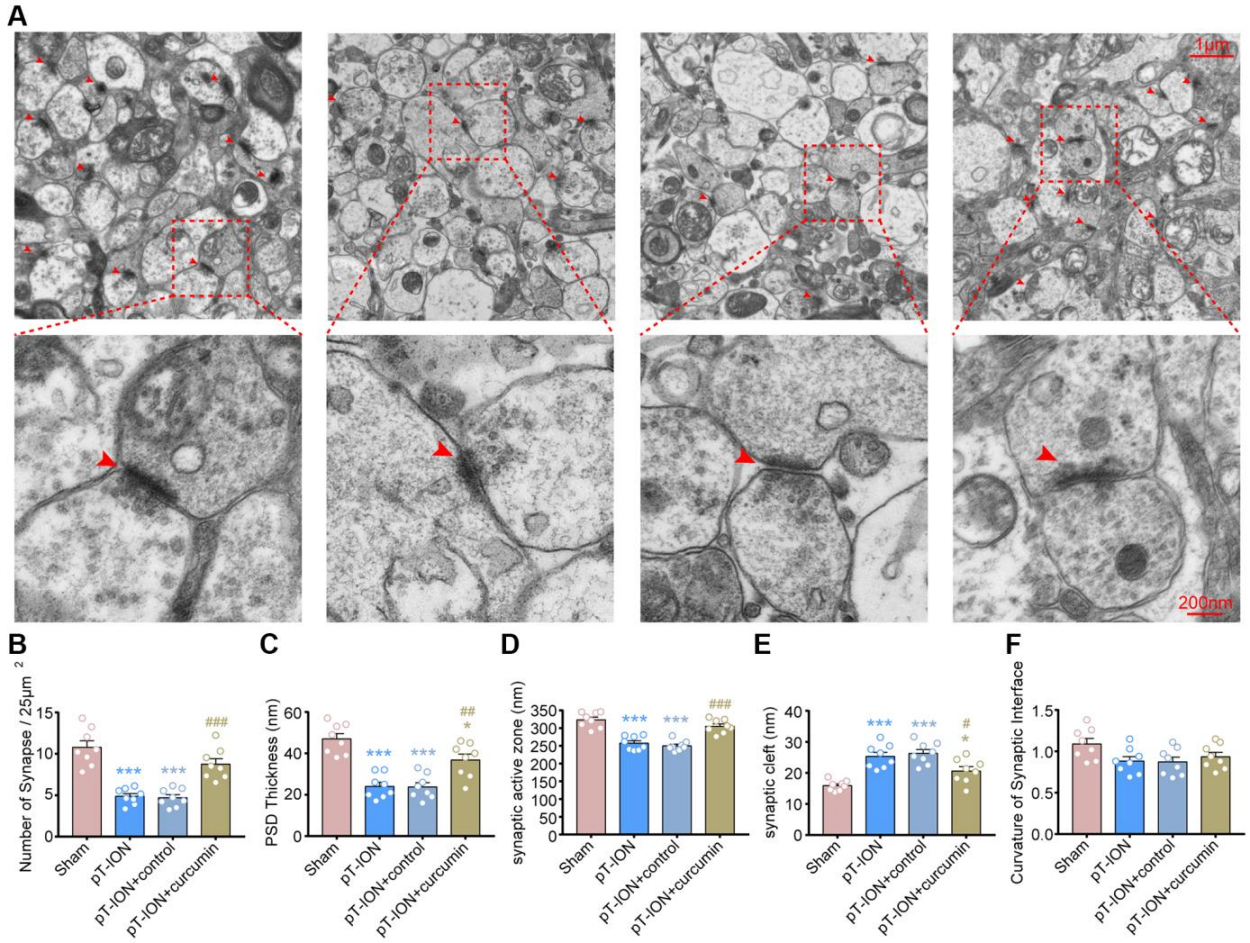
**The protective effects of curcumin on the density and ultrastructure of synapses of hippocampal CA1 region in pT-ION mice.** (**A**) Representative TEM images showing the density and ultrastructure of synapses of hippocampal CA1 region in each group (scale bar: 1 μm and 200 nm). Bar graphs showing the number of synapses/25 μm^2^ (**B**), the thickness of the PSD (**C**), the length of the synaptic active zone (**D**), the width of the synaptic cleft (**E**), and the curvature of the synaptic interface (**F**) in each group. *N* = 8 mice/group. ^*^P < 0.05 and ^***^P < 0.001 vs. sham group; ^#^*P* < 0.05, ^##^*P* < 0.01 and ^###^*P* < 0.001 vs. pT-ION group, one-way ANOVA and Tukey’s test.

We observed the effect of curcumin on the synaptic ultrastructure of the hippocampal CA1 region using TEM (Figure 6A). The results showed that compared with the sham-operated group, the pT-ION group showed a significant decrease in synaptic density (one-way ANOVA and Tukey’s test, *P* < 0.0001, Figure 6B), thickness of PSD (one-way ANOVA and Tukey’s test, *P* < 0.0001, Figure 6C), and length of synaptic active zone (one-way ANOVA and Tukey’s test, *P* < 0.0001, Figure 6D), but had increased width of synaptic clefts (one-way ANOVA and Tukey’s test, *P* < 0.0001, Figure 6E) in the hippocampal CA1 region. This suggests that chronic neuropathic pain causes a structural damage to the synapses of hippocampal CA1 and a decrease in synaptic transmission efficacy. After curcumin treatment, the density of synapses (one-way ANOVA and Tukey’s test, *P* = 0.0003, Figure 6B), thickness of PSD (one-way ANOVA and Tukey’s test, *P* = 0.0049, Figure 6C), and length of synaptic active zone (one-way ANOVA and Tukey’s test, *P* = 0.0001, Figure 6D) significantly increased, while the width of synaptic clefts significantly decreased (one-way ANOVA and Tukey’s test, *P* = 0.0425, Figure 6E), compared with the pT-ION group. However, there were no significant changes in synaptic interface curvature in each group (one-way ANOVA and Tukey’s test, *P* > 0.05, Figure 6F). It was suggested that pT-ION induced structural damage to the synapses of hippocampal CA1, and curcumin could regulate synaptic activity and alleviate the impairment of cognitive functions, such as learning and memory, with good effects.

## DISCUSSION

In the current study, we found that curcumin treatment attenuated pT-ION-induced orofacial injurious hypersensitivity reactions and associated cognitive deficits. Mechanistically, curcumin significantly inhibited the decreased density of total and mushroom-type dendritic spines in the hippocampal CA1. Meanwhile, the decreased synaptic density, PSD thickness, synaptic active zone, and increased synaptic cleft induced by pT-ION were inhibited by curcumin. Together, these findings support our hypothesis that curcumin alleviates orofacial nociceptive hypersensitivity and improves cognitive impairment by regulating the structural synaptic plasticity of hippocampal CA1 in a mouse model of trigeminal neuralgia.

The mouse model of trigeminal neuralgia was established by pT-ION, per our previously established methodology [24]. In the extant literature, chronic constriction injury (CCI) of the ION [25, 26] and cobra venom-induced models [27] are widely used. However, the precision of CCI surgery is difficult to standardize. Further, leakage of cobra venom may also confuse researchers due to the disturbances of off-target nerves [28]. In comparison, the pT-ION model showed good precision and stability [29]. Herein, we partially transected ION to induce orofacial allodynia. In this study, the pT-ION models exhibited sustained and stable orofacial allodynia from post-surgical days 14 to day 28, which is consistent with our previous observations [23, 30] and others [31].

Our results indicate that chronic neuropathic pain induced by pT-ION can also evoke impairment of cognitive functions, such as spatial learning and memory dysfunction. This is consistent with the results of a previous rat model of trigeminal neuralgia induced by cobra venom [12]. Cognitive deficits, especially memory loss, are among the most common complaints of patients with neuropathic pain [32]. Deficits in mental flexibility, working memory, and sustained attention have also been observed in patients with chronic pain, seriously affecting their quality of life. [33, 34]. In parallel with human studies, similar manifestations can also be observed in rodent models [35]. Pincus and Morley [36] emphasized the interactions, rather than one-sided reactions between chronic pain and cognitive impairment. Thus, treatment of cognitive impairment is important for the management of chronic pain. For patients with comorbidities, seeking co-treatment drugs may have better therapeutic effects.

Curcumin has been shown to possess multiple therapeutic properties, such as anti-inflammatory, neuroprotective effects, radioprotective, and anticancer effects [11]. In this study, curcumin treatment increased abnormal orofacial pain thresholds, decreased escape latency and distance to the target platform, and increased time to reach the target area and number of platform crossings in mice. These results suggest that curcumin intervention not only attenuated orofacial allodynia but also alleviated the cognitive impairment induced by pT-ION, consistent with previous observations. Kou et al. found that curcumin can improve cognitive function by modulating endoplasmic reticulum stress under neuroinflammatory conditions [37]. Tiwari et al. reported that curcumin can stimulate neurogenesis by inducing the activation of the Wnt/β-catenin pathway, thereby improving cognitive impairment [8]. In SNI mouse models, the administration of curcumin alleviated mechanical and cold allodynia by inhibiting the JAK2-STAT3 cascade [38]. Therefore, curcumin may be a potential treatment option for patients with chronic neuropathic pain associated with cognitive dysfunction.

Synaptic plasticity was first demonstrated in the hippocampus by Bliss and Lømo [39] and is an integral part of modulating pain experience, memory consolidation, and cognitive functions [40]. Synaptic plasticity is described in both structural and functional forms. However, the study of structural synaptic plasticity, along with its correlated mechanisms of chronic pain, is not as sufficient as functional ones [21]. The present study considered structural synaptic plasticity as the entry point of the mechanism study. Dendritic spines are actin-rich neuron-derived protrusions, wherein these tiny protrusions can modulate synaptic plasticity via reshaping synaptic interface mediated by actin filaments [41]. Changes in dendrite spine density can alter synaptic plasticity, and changes in synaptic plasticity can regulate learning and memory. The formation of new dendritic spines contributes to long-term memory [42]. One study revealed that chronic sleep restriction could induce shortening and shedding of CA1 dendritic trees, which could be notably protected by curcumin [43]. Our previous study indicated that one of the mechanisms of electroacupuncture in the treatment of trigeminal neuralgia and related anxiety-like emotions is to increase the density of dendritic spines in the hippocampus CA1 region [30]. In our study, Golgi staining results showed that the decreased density of dendritic spines and the proportion of mushroom-type dendritic spines in hippocampal CA1 induced by pT-ION were partially reversed by curcumin. The morphology and density of dendritic spines in the hippocampal CA1 region may be the targets of curcumin in the treatment of chronic neuropathic pain and related cognitive impairment.

The density and structure of synapses are also important factors for synaptic plasticity. The PSD thickness, length of the synaptic active zone, and width of the synaptic cleft are sensitive indices of synaptic plasticity. They play key roles in synaptic functions and nerve signal transmission. The increase in the length of the synaptic active zone, PSD thickness, and decrease in the width of the synaptic cleft indicate the enhancement of the synaptic transmission function [44]. Our previous research demonstrated that pT-ION induced a decrease in the density of synapses, length of the synaptic active zone, PSD thickness, and an increase in the width of the synaptic cleft in hippocampal CA1 [30]. The current findings further confirm these results. Moreover, these changes in synaptic plasticity could be reversed by curcumin. These results are consistent with previous results in rat models of cobra venom-induced trigeminal neuralgia [12]. However, we did not observe any changes in the curvature of the synaptic interface after modeling or curcumin intervention although this still needs to be verified in future studies.

Although our study explored possibilities for curcumin treatment of chronic pain and cognitive impairment, the limitations of our study should be mentioned. First, the present study focused on only structural synaptic plasticity and did not study the function of synapses. LTP is a crucial component of functional synaptic plasticity. Du et al. revealed that reversal of LTP in the hippocampal CA1 could improve cognitive function by inhibiting GluA2-containing α-amino-3-hydroxy-5-methyl-4-isoxazole propionic acid receptor endocytosis [45]. Therefore, whether curcumin can repair LTP in the hippocampal CA1 should be the focus of our future research. Second, to date, objective indicators for pain detection are still lacking. Pain is described as “an unpleasant sensory and emotional experience” [46], which reveals its subjectivity. In the clinical context, self-reporting is the most commonly used method for the diagnosis of pain, and it is difficult to accurately describe the intensity of pain. To evaluate pain objectively, we used a blinded method in our experiment. Next, we will explore more objective methods to evaluate pain, particularly medical imaging methods. Functional magnetic resonance imaging (fMRI) technology provides a new dimension in pain assessment [47]. fMRI has been used in clinical trials and animal studies related to pain [48, 49]. These limitations should be considered in future studies.

## CONCLUSION

In the current study, we found that infraorbital nerve injury induced trigeminal neuralgia and cognitive impairment in mice. Curcumin attenuated the orofacial pain and related cognitive impairments induced by pT-ION. These potential mechanisms may be related to the modulation of synaptic plasticity in the hippocampal CA1 region, including the density and structure of dendritic spines and synapses. This indicates the promising therapeutic effects of curcumin on TN and highlights the importance of synaptic plasticity of hippocampal CA1 in pain relief and cognitive restoration.
